# Leukemoid reaction in a patient with metastatic renal cell carcinoma—rare clinical presentation seen in Nepal: a case report

**DOI:** 10.1097/MS9.0000000000000513

**Published:** 2023-04-12

**Authors:** Pramit Khatiwada, Girendra Yadav, Prashant Guragain, Pradip Raut, Viplaw Subedi, Soniya Dulal

**Affiliations:** B.P. Koirala Institute of Health Sciences, BPKIHS, Sunsari, Nepal

**Keywords:** case report, leukemoid reaction, Nepal, poor prognosis, renal cell carcinoma

## Abstract

Leukemoid reaction (increase in leucocyte count >50 ×10^9^ cell/l) occurs due to reactive causes of bone marrow and is diagnosed after excluding the malignant haematological disorder. Leukemoid reaction is a rare clinical presentation in metastatic renal cell carcinoma and is said to have a rare prognosis. This case has had been reported in the line of SCARE criteria.

**Case presentation:**

A case of a 35-year-old female with no known previous co-morbidities presented with a history of abdominal pain in the right flank region for 2 months, fever and cough for 2 months. Physical examination showed palpable mass and tenderness in the right flank and investigations showed leukemoid reaction in peripheral blood smear. The patient was initially treated with strong intravenous antibiotics with suspicion of pyelonephritis in another centre, despite which the patient still had elevated leucocyte count and referred to our centre, where the patient was evaluated for elevated leucocyte count and with further investigations, ruled out any malignant haematological disorder. Final diagnosis of renal cell carcinoma was made by renal mass biopsy. The patient underwent targeted therapy with sunitinib. The patient expired and further investigation and follow-up were not possible.

**Conclusion:**

The lack of data and evidence of extensive diagnostic tests is the reason we are unable to assume leukemoid reaction as a poor prognostic factor in case of metastatic renal cell carcinoma. The presence of other paraneoplastic syndromes with renal cell carcinoma might have resulted in the poor prognosis that cannot be excluded.

## Introduction

HIGHLIGHTS

- Leukemoid reaction (increase in leucocyte count >50 ×10^9^ cell/l) occurs due to reactive causes of bone marrow and is diagnosed after excluding the malignant haematological disorder.
- Leukemoid reaction is a rare clinical presentation in metastatic renal cell carcinoma (RCC).
- Granulocyte-colony stimulating factor (G-CSF) plays a role in the proliferation and differentiation of hematopoietic progenitor cells.
- Leukemoid reaction is said to have a poor prognosis in metastatic RCC.

Leukemoid reaction is defined as an increase in leucocyte count of more than 50×10^9^ l in the absence of myeloproliferative disorders, such as chronic myelogenous leukaemia and chronic neutrophilic leukaemia 1. Leukemoid reaction is a rare clinical presentation in metastatic RCC and is said to have a rare prognosis^2^.

## Methodology

This case has had been reported in the line of SCARE criteria^3^.

## Case presentation

A case of a 35-year-old female with no known previous co-morbidities presented with a history of abdominal pain in the right flank region for 2 months, fever and cough for 2 months. Physical examination showed palpable mass and tenderness in the right flank. Her Eastern Cooperative Oncology Group (ECOG) performance status score^4^ was 3. Initially, she was treated in another secondary centre in the line of pyelonephritis with antibiotics and was referred to our centre, BPKIHS, for further evaluation and management because of non-improving symptoms despite medical management with antibiotics.

In our centre, she went through a series of following investigations. Her complete blood count showed a total leucocyte count of 10^10^ cells/l. The septic workup, blood culture, and urine culture were sterile. Her peripheral blood smear, bone marrow aspiration, and biopsy were sent to rule out any myeloproliferative disorder. Bone marrow aspiration and biopsy report shows normal findings and is non-suggestive of any myeloproliferative disorders. Her peripheral blood smear showed a leukemoid reaction.

**Figure 1:**
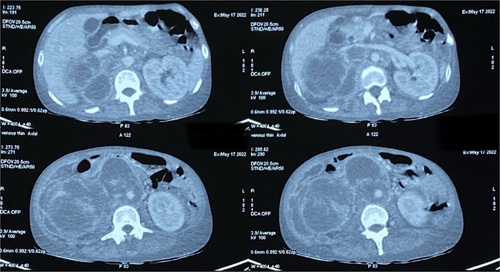
CECT report showing right renal mass. CECT, contrast enhanced computed tomography

**Figure 2:**
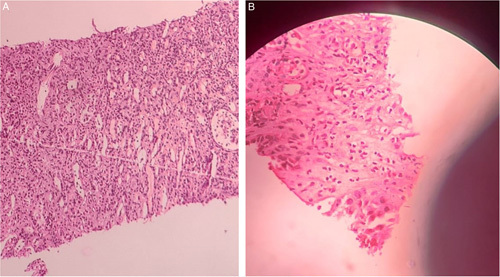
(A) Renal cell carcinoma confirmed by pathological examination. (B) Magnified view of renal cell carcinoma confirmed by pathological examination.

Ultrasonography of abdomen and pelvis was done which showed enlarged right kidney (12×7 cm in size) with heterogeneous echotexture, dilated upper pole calyces with internal echoes within. Rest of the pelvi-calyceal system was not dilated. Her chest X-ray showed the presence of bilateral opacities in all of the lung fields. High resolution computed tomography chest showed multiple cannonball metastasis. Her contrast enhanced computed tomography chest abdomen and pelvis report showed a mass of 15×14×8 cm right renal mass with lung metastases (Fig. 1). Biopsy from renal lump showed RCC (Fig. 2). She received targeted therapy of sunitinib (Tab. SUNITINIB 50 mg PO OD for 4 weeks then 2 weeks off). However, the patient lost to follow-up. She was reported dead after around a month of diagnosis, cause of which cannot be ascertained.

## Discussion and conclusion

Leukemoid reaction occurs due to reactive causes of bone marrow and is diagnosed after excluding the malignant haematological disorder. The major causes of leukemoid reactions are severe infections, intoxications, malignancies, severe haemorrhage, or acute haemolysis^5^ In our case, the patient was initially treated with strong intravenous antibiotics with a suspicion of pyelonephritis in another centre , despite which the patient still had elevated leucocyte count. In our centre, the patient was evaluated with further investigation which ruled out malignant haematological disorder and final diagnosis of RCC was made by renal mass biopsy. Presence of leukemoid reaction in solid tumours like RCC is very rare^2^. This forms the basis for reporting this case.

G-CSF plays a role in the proliferation and differentiation of hematopoietic progenitor cells. Several non-haematologic malignancies like genitourinary, lung, skin, gastrointestinal, and oropharyngeal origin producing G-CSF presenting with leukemoid reaction have been reported^6^. Prognosis in such case of leukemoid reaction was very poor^7^–^10^. Assessment of the level of G-CSF could not be done in our case because it was not available in our centre.

However, the presence of other paraneoplastic syndromes with RCC might have resulted in the poor prognostic factor cannot be excluded. An extensive investigation and follow-up are missing in this case due to the unavailability of modern tests in our centre. The patient expired and further investigation and follow-up were not possible. The lack of data and evidence of extensive diagnostic tests, which is necessary to assume leukemoid reaction as a poor prognostic factor in cases of metastatic RCC, indicates the need for further study^10^.

## Ethical approval

Not applicable

## Consent

Written informed consent was taken from patient for publication of this case report and also for any accompanying images. A copy of written consent is available for review by the Editor-in-Chief of this journal on request.

## Sources of funding

No sources of funding.

## Conflicts of interest disclosure

All authors declare that they have no conflicts of interest.

## Guarantor

Pramit Khatiwada

## Provenance and peer review

Not commissioned, externally peer-reviewed.
